# Theory of planned behaviour can help understand processes underlying the use of two emergency medicine diagnostic imaging rules

**DOI:** 10.1186/s13012-014-0088-x

**Published:** 2014-08-07

**Authors:** Richard Perez, Jamie C Brehaut, Monica Taljaard, Ian G Stiell, Catherine M Clement, Jeremy Grimshaw

**Affiliations:** Ottawa Hospital Research Institute, Clinical Epidemiology Program, Ottawa, ON Canada; Department of Epidemiology & Community Medicine, University of Ottawa, Ottawa, ON Canada; Department of Medicine, University of Ottawa, Ottawa, ON Canada; Department of Emergency Medicine, University of Ottawa, Ottawa, ON Canada

**Keywords:** Clinical decision rules, Canadian C-Spine Rule, Canadian CT-Head Rule, Theory of planned behaviour, Emergency physicians, Implementation study

## Abstract

**Background:**

Clinical decision rules (CDRs) can be an effective tool for knowledge translation in emergency medicine, but their implementation is often a challenge. This study examined whether the Theory of Planned Behaviour (TPB) could help explain the inconsistent results between the successful Canadian C-Spine Rule (CCR) implementation study and unsuccessful Canadian CT Head Rule (CCHR) implementation study. Both rules are aimed at improving the accuracy and efficiency of emergency department radiography use in clinical contexts that exhibit enormous inefficiency at the present time. The rules were prospectively derived and validated using the same methodology demonstrating high sensitivity and reliability. The rules subsequently underwent parallel implementations at 12 Canadian hospitals, yet only the CCR was observed to significantly reduce radiography ordering rates, while the CCHR failed to have any significant impact at all. The drastically different results are unlikely to be the result of differences in implementation strategies or the decision rules.

**Methods:**

Physicians at the 12 participating Canadian hospitals were randomized to CCR or CCHR TPB surveys that were administered during the baseline phases of the implementation studies, before any intervention had taken place. The collected baseline survey data were linked to concurrent baseline physician and patient-specific imaging data, and subsequently analyzed using mixed effects linear and logistic models.

**Results:**

A total of 223 of the 378 eligible physicians randomized to a TPB survey completed their assigned baseline survey (CCR: 122 of 181; CCHR: 101 of 197). Attitudes were significantly associated with intention in both settings (CCR: ß = 0.40; CCHR: ß = 0.30), as were subjective norms (CCR: ß = 0.26; CCHR: ß = 0.73). Intention was significantly associated with actual image ordering for CCR (OR = 1.79), but not CCHR.

**Conclusions:**

The TPB can be used to better understand processes underlying use of CDRs. TPB constructs were significantly associated with intention to perform both imaging behaviours, but intention was only associated with actual behaviour for CCR, suggesting that constructs outside of the TPB framework may need to be considered when seeking to understand use of CDRs.

**Electronic supplementary material:**

The online version of this article (doi:10.1186/s13012-014-0088-x) contains supplementary material, which is available to authorized users.

## Background

In environments where use of health resources is inefficient and busy physicians have little time to consider and adopt complex new guidelines, clinical decision rules (CDRs) can be a highly successful vehicle for knowledge translation (KT). CDRs help physicians making specific, high volume decisions by providing a simple algorithm based on a small number of highly diagnostic, easily accessible indicators identified through original research [1,2]. When used widely and appropriately, CDRs can reduce practice variation, improve patient experience with the healthcare system, and save healthcare resources without sacrificing safety [2–4]. For example, implementation studies of a variety of rules for imaging decisions have already been demonstrated to result in important reductions in imaging rates [5–9].

Whereas properly developed and validated CDRs have great potential to improve patient care, there is much to be learned about how to implement such KT interventions effectively [4,10]. This was clearly demonstrated through the experience of two recently developed rules: the Canadian C-Spine Rule (CCR) [5] and the Canadian Computed Tomography Head Rule(CCHR) [11]. Developed in Ottawa by the same team at approximately the same time, both rules target high-volume, high-severity injuries in the ED – injuries that, prior to rule development, would have resulted in a large proportion of negative imaging that display no evidence of any important adverse clinical conditions. Parallel derivation and validation studies [12,13] showed that both rules provided a clear, clinically acceptable and valid approach to imaging decisions that could reduce rates of imaging without sacrificing patient safety and satisfaction. Subsequently, parallel intervention studies using similar methods in identical pairs of hospitals assessed the extent to which low-cost interventions (*i.e*., physician education and buy-in sessions, distribution of pocket cards and posters, and a mandatory reminder at the point of image requisition) could reduce rates of image requests. Despite the similarity between the interventions, the rules and their development process, results of the intervention studies were markedly different: whereas C-Spine imaging rates were shown to decrease significantly as a result of the intervention [5], CT Head imaging rates actually increased during the intervention period [11].

**Figure 1:**
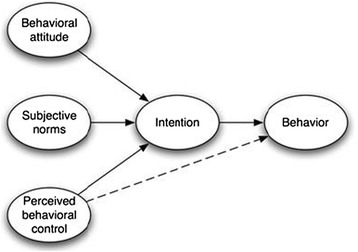
**Theory of planned behaviour model** [17]**.**

These different results stemming from two seemingly similar interventions suggest that we do not understand the causal pathways that underlie successfully changing physician image ordering behaviours. In the broader KT literature, this is common: reviews of the effectiveness of many KT interventions show considerable variability [10,14]. To better understand the potential pathways by which health provider behaviours can be changed to improve healthcare, increasing attention is being paid to developing a theoretical understanding of KT and behaviour change [15,16]. One of the most commonly exploited theories in this context has been the Theory of Planned Behaviour (TPB) [17]. According to the TPB (Figure 1), the primary determinant of an individual’s behaviour is their intention to perform that behaviour, which in turn is a function of attitudes towards the behaviour (whether they feel it is good or bad), subjective norms (whether they perceive important others to support performance of the behaviour), and perceived behavioural control (whether the individual feels the behaviour is under their control) [17]. Across a range of 16 different studies of health provider behaviours, these constructs correlated strongly to changes in target behaviour, on average accounting for 31% of the variability in behaviour, and 59% of the variability in intention to engage in the behaviour [18]. The theory’s extensive use in numerous contexts has led to the development of a well-defined methodology for creating TPB-related tools [19].

As part of the design of the CCR and CCHR intervention studies discussed above, we undertook an exploration of whether TPB constructs could shed light on our efforts to change decision-making behaviours among emergency physicians. For each study, we identified a specific target behaviour that would be the focus of the surveys (‘clinically clearing’ the C-Spine, *i.e*., managing the C-Spine patient without imaging, or managing patients without CT Head imaging). Prior to the intervention phase of each study, we conducted surveys of physicians in the study hospitals designed to assess TPB constructs in light of the target behaviour. Our original goals were to examine: a) whether TPB constructs are associated with stated intention to use the CDRs and manage patients without imaging in the context of two clinical decisions; and b) whether TPB constructs are associated with behaviour as measured by ordering rates. These surveys have since taken on a new importance, given the markedly different findings in the two implementation studies: they allow us to examine whether TPB can shed light on the processes underlying imaging decisions in the two contexts.

## Methods

### Cluster randomized trials evaluating implementation of two CDRs

This study was embedded in the baseline phase of both the CCR and CCHR implementation trials. The details of the methods and results of the CCR and CCHR implementation trials have been published elsewhere [5,11,20]. Briefly, both studies were cluster randomized trials involving the same 12 hospitals, which were stratified (teaching vs. community hospitals), and then pair-matched on baseline radiological ordering rates. Intervention hospitals in one study served as control sites in the other. For both studies, the intervention was a suite of implementation activities that included education, hospital policy changes, and CDR reminders on image requisitions. Control sites received no active interventions regarding the target behaviour. Primary outcomes were number of diagnostic images (C-Spine imaging rate, referrals for CT scan of the head) during two 12-month periods (before and after the intervention). Here, we use only data from the 12-month period before the interventions: 5,680 patients in the CCR study with spinal injury, and 1,925 patients with head injury.

### Survey development and administration

Parallel surveys were developed for both the CCR and CCHR studies; questions and response anchors are provided in the Additional files 1 and 2. Each was designed to measure all four TPB constructs (attitude, subjective norms, perceived behavioural control, and intention) in the context of a) clearing the C-Spine without imaging and b) managing patients without CT Head imaging. In accordance with standard methods using the ‘direct measure’ approach [19], each construct was measured with three to five similar closed-ended questions using 7-point numerical response scales. The one exception, a 6-point response scale used in the measurement of intention, had its responses reweighted to a 7-point scale to maintain consistency with other responses. This was achieved by dividing the responses of the 6-point scale item by 6 and then multiplying by 7, thereby providing responses that ranged from 1 to 7. All questions had defined anchors at the extremes. The mean was taken as the summary construct score. Attitude referred to whether the participant felt that management without imaging was worthwhile (*e.g*. ‘Overall, I think clinically clearing the C-Spine is good practice/bad practice’). Subjective norms referred to whether important others valued the management without imaging (*e.g*., ‘Most of my professional colleagues will clinically clear the C-Spine’). Perceived behavioural control referred to whether the physician felt that management without imaging was within their control to do (*e.g*., ‘clinically clearing the C-Spine is easy/difficult’). Intention addressed whether the physician intended to engage in the behaviour (*e.g*., ‘I intend to clinically clear the C-spine’).

In addition to the TPB construct questions, we asked demographic and practice questions including physician’s sex, year of birth, year of medical school graduation, employment status (full time/part time), years of work in emergency medicine, medical credentials, and average number of hours worked in emergency medicine each week. Surveys were initially pilot-tested among the investigator group, and then on two emergency department physicians from the target population. The final surveys were two pages in length and could be completed in less than 10 minutes.

All active emergency physicians practicing at each of the 12 hospitals were identified as eligible for the surveys and were randomly assigned, using Excel’s RAND function, to receive either a CCR or CCHR survey. Physicians were approached for participation in their assigned survey by site coordinators during the baseline phase of the implementation studies. A cover letter described the nature and purpose of the study, informed physicians that their participation was voluntary, and indicated that the study had been approved by the Ottawa Hospital Research Ethics Board. Completion and return of the survey to the study nurse served as tacit consent. Non-responders were given a second survey, either in person or in their ED mailbox as appropriate.

### Analysis

Descriptive statistics described the distribution of demographic and practice variables for the two surveys, and simple bivariable statistics (*t*-test, Chi-Squared test) were used to identify any differences between them.

After survey data collection but before analysis of the survey data, we linked physician-specific survey data with summary image ordering data prospectively collected as part of the baseline phases of the primary studies. Physicians who completed the CCR survey had their survey data (TPB construct scores) linked to each of their corresponding CCR patient cases; those completing CCHR surveys were linked to their CCHR cases. Patient-specific information included whether an image was sought (yes/no), the identification code of the attending physician, the hospital where the patient was treated, whether the patient arrived by ambulance (yes/no), and whether the patient was admitted to the hospital (yes/no).

The ability of the TPB to explain variance with respect to management without imaging was examined in two ways: association with intention to engage in the target behaviour; and association with the actual target behaviour itself. Most commonly, TPB-based studies have been used in contexts where actual behaviour of health professionals has not been measured, and where stated intention to engage in the behaviour is used as the proxy outcome [18]. In such situations, the most common modeling approach is to use multiple regression to examine the extent to which attitudes, subjective norms, and perceived behavioural control are associated with stated intention. However, it is now well-known that stated intentions to engage in a behaviour often do not correspond to actual behaviour; there is an ‘intention-behaviour gap [21]. In the far smaller number of TPB health provider studies that include actual measures of behaviour (16 of 78 studies [22]), most examined the role of TPB constructs by evaluating the association between the two constructs proposed to be proximal to behaviour (perceived behavioural control and intention) and the target behaviour. We adopted both of these techniques in the current work.

We first measured the association between TPB constructs and intention to manage without imaging (CCR or CCHR) using linear mixed-effects regression models. Intention to clinically clear without imaging was the dependent variable and attitude, subjective norms, and perceived behavioural control were included as fixed effects. Hospitals were declared as random effects to account for intracluster correlation of survey responses within hospitals. Models were estimated using Restricted Maximum Likelihood with Kenward-Roger degrees of freedom.

We also examined the association between TPB constructs and actual behaviour, *i.e*., management without imaging using mixed effects logistic regression models. Radiography decision (no/yes) was specified as the dependent variable, and intention and perceived behavioural control were included as fixed effects. Physician and hospital identifiers were entered as random effects to account for clustering of patients by physician and hospital. To account for potential differences in the patient case-mix among hospitals and physicians, the models adjusted for two severity-related covariates as fixed effects in the models: arrival by ambulance, and hospital admission. All analysis was performed using SAS Version 9.2.

## Results

**Figure 2:**
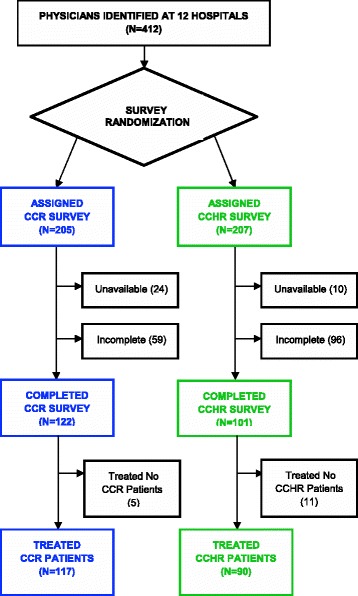
**TPB-Survey Administration within CDR implementation studies.**

Figure 2 describes the distribution of the different surveys across the two implementation studies. A total of 412 emergency medicine physicians were identified as registered for practice within the 12 study hospitals. Of the 205 physicians randomly allocated to receive the CCR survey, 24 were not active at the time of their survey administration and were therefore excluded. Of the 207 physicians randomly allocated to receive the CCHR survey, 10 were not active and were excluded. Among the remaining physicians, 67% (122 of 181) physicians allocated to the CCR, and 51% (101 of 197) allocated to the CCHR survey completed their surveys. For the analyses of imaging behaviours, a further 5 CCR physicians and 11 CCHR physicians were excluded as they contributed no corresponding patients during the baseline phase.

Table 1 describes demographic and practice details, mean TPB construct scores, and the distributions of patients among physician survey respondents. CCR and CCHR respondent samples were similar on all demographic and practice details (p >0.05 in all cases). TPB construct mean scores were above 4.0 (*i.e*., all on the high side of the 7-point scale), generally indicating high scores on intentions, attitudes, norms and control towards management without imaging. Mean intention (t = 7.02, p <0.001), attitude (t = 7.11, p <0.001), and subjective norm (t = 6.09, p <0.001) scores were significantly higher for CCR than for CCHR; mean scores did not differ for perceived behavioural control (t = 1.88, p = 0.06). Patient caseload was higher for CCR physicians (mean caseload 19.3, range 1 to 61) than for CCHR physicians (mean caseload 6.0; range 1 to 26) (t = 8.67, p <0.001), indicating higher prevalence of CCR injuries in the population. A greater proportion of CCR than CCHR patients were cleared without imaging (CCR 45.0%; CCHR 33.3%; *χ*^2^ = 24.8; p <0.001).

**Table 1:** **Demographics, summary TPB construct scores and patient caseload from CCR and CCHR study survey respondents**

	**CCR (N = 122)**	**CCHR (N = 101)**	**Comparison (p-value)**
**Demographics**			
Age (mean [SD])	40.5 (8.6)	39.5 (9.1)	t_(206) =_ 1.89 (0.40)
Male (% (n))	79.3 (96)	79.2 (80)	t_(220)_ = −0.02 (0.98)
Years since Medical School	13.7 (9.2)	13.0 (8.1)	t_(217)_ = 0.66 (0.51)
Graduation (mean [SD])			
Years Practicing Emergency Medicine (mean [SD])	10.6 (8.4)	9.3 (7.7)	t_(102)_ = 1.05 (0.30)
Hours per Week Treating ED Patients (mean [SD])	27.0 (12.1)	29.9 (9.5)	t_(178)_ = −1.70 (0.91)
Emergency Credentials (%, [n])			
CCFP	10.7 (12)	10.4 (10)	t_(206)_ = 0.07 (0.95)
CCFP (EM)	45.5 (51)	45.8 (44)	t_(206)_ = −0.04 (0.97)
FRCPC	43.8 (49)	42.7 (41)	t_(206)_ = 0.15 (0.88)
Dip ABEM	14.3 (16)	11.5 (11)	t_(206)_ = 0.60 (0.55)
**TPB Construct Scores** (mean [SD])			
Intention	6.39 (0.68)	5.30 (1.54)	t_(221)_ = 7.02 (<0.001)
Range	(4.2-7.0)	(1.0-7.0)	
Attitude	6.37 (0.91)	5.22 (1.47)	t_(221)_ = 7.11 (<0.001)
Range	(2.3-7.0)	(1.0-7.0)	
Subjective Norms	5.93 (0.86)	5.02 (1.35)	t_(221)_ = 6.09 (<0.001)
Range	(3.5-7.0)	(1.0-7.0)	
Perceived Behavioural Control	5.00 (0.61)	4.85 (0.59)	t_(221)_ = 1.88 (0.06)
Range	(3.6-6.6)	(2.6-6.2)	
**Patient caseload among survey**			
**Respondents**			
Physicians with Patient Cases	117	90	
Range of Patient Caseload	1-61	1-26	
Mean Patient Caseload	19.3	6.0	t_(205)_ = 8.67 (<0.001)
Imaging Rate (% [SD])	55.6 (0.2)	66.4 (0.3)	t_(205)_ = −2.90 (0.004)
Physicians without Patient Cases	5	11	

### Intention to manage without radiography

Table 2 describes the association between the TPB constructs and intention to manage without imaging for both C-Spine and CT imaging decisions. In the CCR model, attitude and subjective norms were significantly associated with intention: the mean increase in intention was 0.40 out of 7 (95% CI: 0.29 to 0.50) for every unit increase in attitude scores, 0.26 out of 7 (95% CI: 0.15 to 0.38) for every unit increase in subjective norm scores. Perceived behavioural control was not significantly associated with intention (p = 0.35). Overall, the TPB constructs explained 56% of the variance in reported intentions to clinically clear the C-Spine.

**Table 2:** **Mixed linear models evaluating the association between TPB constructs and intention to manage without radiography**

**Survey**	**CCR (N = 122)**			**CCHR (N = 101)**		
**Variables**	**Parameter estimate (95% CI)**	**S.E.**	**P-value**	**Parameter estimate (95% CI)**	**S.E.**	**P-value**
Attitude	0.40 (0.29, 0.50)	0.05	<0.001	0.30 (0.16, 0.45)	0.07	<0.001
Subjective Norms	0.26 (0.15, 0.38)	0.06	<0.001	0.73 (0.57, 0.88)	0.08	<0.001
Perceived Behavioural Control	−0.07 (−0.21, 0.07)	0.07	0.354	0.15 (−0.12, 0.41)	0.13	0.273

The CCHR model showed similar results. Attitude and subjective norms scores were significantly associated with intention scores: the mean increase in intention was 0.30 out of 7 (95% CI: 0.16 to 0.45) for every unit increase in attitude scores, and 0.73 out of 7 (95% CI: 0.57, 0.88) for every unit increase in subjective norms scores. Again, perceived behavioural control was not significantly associated with intention (p = 0.27). Overall, the TPB constructs explained 81% of the variance in reported intentions to manage without CT imaging.

### Actual management without radiography

The results of the two mixed effects logistic regression models to evaluate the association between the TPB constructs and actual management without imaging are presented in Table 3. The CCR model showed intention to be significantly associated with imaging decisions (OR = 1.79; 95% CI 1.40 to 2.29); a 79% increase in odds of management without imaging was associated with each unit increase in intention scores. Perceived behavioural control was not significantly associated with imaging decisions (OR = 0.79; 95% CI 0.60 to 1.03). The CCHR model showed markedly different results: neither of the TPB constructs (intention, perceived behavioural control) were significantly associated with imaging decisions (intention: OR = 1.05; 95% CI 0.87 to 1.28); (perceived behavioural control: OR = 0.92; 95% CI 0.57 to 1.50). Patient severity indicators were significant in both models: patients who arrived by ambulance or who were admitted to the hospital were less likely to be clinically cleared without imaging.

**Table 3:** **Mixed-effects logistic regression models evaluating the association between TPB constructs and actual management without imaging**

**Survey**	**CCR**			**CCHR**		
	**(Physicians = 117, patients = 2,260, images = 1,242)**			**(Physicians = 90, patients = 544, images = 363)**		
**Variables**	**OR (95% CI)**	**S.E.**	**P-value**	**OR (95% CI)**	**S.E.**	**P-value**
Intention	1.79 (1.40, 2.29)	0.12	<0.01	1.05 (0.87, 1.28)	0.10	0.60
Perceived Behavioural Control	0.79 (0.60, 1.03)	0.13	0.08	0.92 (0.57, 1.50)	0.24	0.74
Arrival by Ambulance	0.24 (0.20, 0.30)	0.10	<0.01	0.63 (0.40, 0.98)	0.23	0.04
Hospital Admission	0.18 (0.09, 0.38)	0.37	<0.01	0.20 (0.10, 0.39)	0.34	<0.01

## Discussion

This study took advantage of a pair of CDR implementation studies at 12 Canadian EDs targeting two image ordering behaviours. Theory-based surveys were designed to determine whether TPB constructs were relevant in an emergency medicine context, and whether we could use these constructs to describe both intention to manage patients without imaging, and their actual management behaviours. With remarkably different results of the two implementation studies (the CCR study showing reduced imaging behaviour as expected, and the CCHR study actually showing an increase), we set out to investigate TPB constructs as possible contributors to these discrepant results. Our findings suggest that the TPB model can be usefully applied to emergency medicine imaging decisions: TPB constructs were related to intention to manage patients without imaging, with models explaining 56% and 81% of variability for CCR and CCHR imaging decisions, respectively. Furthermore, we found that intention is significantly associated with actual imaging decisions for C-Spine injuries.

Intention to engage in the behaviour is predicted to be a necessary condition for most KT interventions, including imaging decisions. Our efforts to understand factors associated with intention to manage patients without imaging have been instructive. First, there was variability in measured intention across physicians, suggesting that despite the solid evidence base underlying use of the rules to manage patients without imaging, not all physicians strongly intended to use them. Second, efforts to change those intentions are at least partly related to TPB constructs. Both CCR and CCHR imaging models showed that intention was associated with attitude and subjective norms, but not with behavioural control (*i.e*., the extent to which a physician feels they have control over managing patients without imaging). The results suggest that in an environment similar to emergency medicine where physicians tend to practice with a great degree of autonomy (thus control over the behaviour is not an issue), future implementation efforts seeking to improve intention may focus on changing the attitudes of the providers and those around them. The potential improvement, however, could be limited if baseline values are already relatively high, as demonstrated in the contexts of CCR and CCHR decisions of this study. In such circumstances, constructs outside of the TPB framework may prove more effective targets in changing behaviour.

Our analyses of the theoretical predictors of actual imaging were also instructive, particularly in the context of the differing results of the two implementation studies. Recall that the CCR intervention study was effective in reducing imaging rates after the intervention. Our theory-based survey showed that in the context of these CCR decisions, intention was strongly associated with imaging behaviour (and again, perceived behavioural control was unrelated to that behaviour). In the context of the CCHR intervention study, results were very different. Not only was the intervention ineffective in changing imaging, but the parallel theory-based survey mirrored these results by showing that TPB constructs (intention, perceived behavioural control) were unrelated to imaging behaviour. Given the significantly lower mean baseline values of TPB constructs in the CCHR context, one may have expected a priori for there to be greater opportunity to improve CCHR imaging decisions through changes in the TPB constructs. From a theoretical point of view, this suggests that factors that governed CCHR imaging behaviour may lie outside of the TPB framework. A number of such factors have been proposed, including overcrowding in emergency departments, increased access to CT imaging, a trend towards greater use of CT imaging, as well as cognitive mechanisms that may operate regardless of intention, such as habit and difficulty remembering the rule [11,23,24]. While some of these factors could be argued to be indirectly related to TPB constructs, none fall easily within the TPB framework. For example, difficulty remembering information may not be explicitly noticed or acknowledged by the provider [4], and therefore may not be related to either to perceived behaviour control or intention. These results suggest that the TPB, while a good starting point, may not address all important elements of implementing KT in emergency medicine. Current efforts to develop frameworks that assess the impact of other important determinants of behaviour [22,25,26] should be further developed in this context. Volitional constructs such as implementation intentions and attention control have been successfully appended to TPB in other areas to better explain the intention-behaviour gap, and could be the next important step in furthering our understanding of CDR use [27,28].

### Strengths

This project has a number of specific strengths. Since we had access to both survey and imaging data, we were able to evaluate both TPB predictors of intention and actual behaviour; many studies evaluate intention without measuring the actual behaviour of healthcare professionals [18]. The lack of association between intention and management without CT imaging in this study illustrates how measuring intention alone can be inadequate if our goal is to understand behaviour. Furthermore, in contrast to many studies that examine only self-reported behaviour, our study included objective behavioural data on imaging decisions for thousands of patients. Finally, our complex hierarchical analyses (accounting for clustering by hospital and physician) allowed us an appropriate level of statistical control in our analysis, and to avoid spurious findings.

### Limitations

This study has limitations that warrant consideration. We did not conduct a detailed examination of the reliability or validity of the TPB in the current context, instead choosing to rely on standardized TPB survey methodology that has been developed elsewhere and applied in many contexts [19]. Only direct measures of TPB constructs were used because we did not have the resources to collect indirect measures. The analysis also only used baseline measures of the TPB constructs and behaviour, before the interventions were actually implemented. Physicians treated fewer CCHR than CCR patients in the original implementation studies, resulting in smaller CCHR patient sample sizes, which gave us reduced power to detect associations between the TPB constructs and CCHR decisions. We were unable to assess responder bias as we did not have detailed demographic or practice detail information on non-responders. As a result, we cannot be sure that the sample of respondents did not differ in important ways from the larger population of physicians at these 12 hospitals. Furthermore, TPB construct means were near ceiling in many cases, particularly for the CCR study; however, such ceiling effects would likely only have attenuated any of the effects already identified. Finally, while we considered doing a structural equation model analysis involving all proposed TPB pathways within a single model, we could not do this and still account for the clustering by hospital and patient.

## Conclusions

This study shows that the Theory of Planned Behaviour framework can be used to better understand processes underlying intention towards and behaviours related to use of clinical decision rules. TPB constructs were significantly associated with intention to behave in a manner consistent with these rules, and may suggest ways of further encouraging such behaviour. While positive intention is an important prerequisite for imaging decisions consistent with CDRs, it is not always sufficient, and our findings suggest that there are important predictors of imaging decisions not currently incorporated by the TPB that need to be explored. Finally, our results shed light on the unexpected results of two previous implementation studies [3]. Despite similar rules, derivation and validation processes, and implementation, the CCR study [5] significantly reduced image ordering rates, while the CCHR study [11] did not. Mirroring these results, our study showed that baseline measures of the TPB constructs were significantly associated with concurrent baseline behaviour for the CCR, but not the CCHR. To explain these findings, the TPB framework focuses our attention on identifying pathways affecting imaging behaviours that are independent of physician intentions. Future work should build on frameworks like the TPB to incorporate such intention-independent processes, and examine the extent to which changes in these constructs result in changes in the target behaviour.

## Additional files

Additional file 1:
**Description of TPB items from CCR surveys, arranged by construct.**
Additional file 2:
**Description of TPB items from CCHR surveys, arranged by construct.**

## Abbreviations

CCHR: Canadian CT-Head rule
CCR: Canadian C-Spine Rule
CDR: Clinical decision rule
KT: Knowledge translation
TPB: Theory of planned behaviour
