# Supplementary material for: Theory of planned behaviour can help understand processes underlying the use of two emergency medicine diagnostic imaging rules
Source: Implement Sci. 2014 Aug 7;9:88. doi: 10.1186/s13012-014-0088-x (PMC4243777; doi:10.1186/s13012-014-0088-x)
Supplement: Additional file 1: — Description of TPB items from CCR surveys, arranged by construct. [file 13012_2014_88_MOESM1_ESM.docx]

Appendix 1. Description of TPB items from CCR surveys, arranged by construct

| **TPB Construct** | **Cronbach’s Alpha** | **Survey Item Descriptions with *Ends of Response Scale*** | **Mean (S.D.)** | **Cronbach’s Alpha if Item Removed** |
| --- | --- | --- | --- | --- |
| Attitude (n = 121) | 0.91 | 1. Clinically Clearing the C-Spine is: *Bad Practice / Good Practice* | 6.53 (0.75) | 0.92 |
|  |  | 2. Clinically Clearing the C-Spine is: *Harmful / Beneficial* | 6.26 (1.19) | 0.87 |
|  |  | 3. Clinical Clearing the C-Spine is: *Negative / Positive* | 6.40 (0.94) | 0.86 |
|  |  | 4. Clinical Clearing the C-Spine is the: *wrong thing to do / right thing to do* | 6.28 (1.21) | 0.85 |
|  | | | | |
| Subjective Norms  (n = 115) | 0.26 | 1. Most of my Professional Colleagues will Clinically Clear the C-Spine: *Definitely No / Definitely Yes* | 5.68 (1.00) | -0.17 |
|  |  | 2. People Important to Me think I should Clinically Clear the C-Spine: *Definitely Should Not/Definitely Should ** | 3.52 (2.05) | 0.69 |
|  |  | 3. The Canadian Association of Emergency Physicians would: *Definitely Disapprove/ Definitely Approve* | 6.12 (0.96) | -0.21 |
|  | | | | |
| Perceived Behavioral Control  (n = 118) | 0.72 | 1. Clinically Clearing the C-Spine is: *Difficult / Easy ** | 4.28 (1.62) | 0.71 |
|  |  | 2. Likelihood that you will be able Clinically Clear the C-Spine is: *Very Unlikely / Very Likely* | 5.69 (1.00) | 0.58 |
|  |  | 3. I am confident that I could Clinically Clear the C-Spine if I wanted to: *Strongly Disagree / Strongly Agree* | 5.91 (1.22) | 0.56 |
|  |  | 4. Factors outside my control prevent me from Clinically Clearing the C-Spine: *Strongly Agree / Strongly Disagree ** | 4.11 (2.03) | 0.65 |
|  |  | 5. How much control do you have over Clinically Clearing the C-Spine: *No Control / Complete Control* | 5.72 (1.12) | 0.52 |
|  | | | | |
| Intention  (n = 120) | 0.92 | 1. I intend to Clinically Clear the C-Spine: *Definitely Do Not / Definitely Do* | 6.54 (0.71) | 0.88 |
|  |  | 2. I want to Clinically Clear the C-Spine: *Definitely Do Not / Definitely Do* | 6.58 (0.72) | 0.88 |
|  |  | 3. I plan to Clinically Clear the C-Spine: *Definitely Do Not / Definitely Do* | 6.52 (0.79) | 0.88 |
|  |  | 4. My Desire to Clinically Clear the C-Spine can be described as: *No Desire / Very Strong* | 5.96 (0.77) | 0.93 |

* Polarity of anchors is reversed relative to anchors of other items used for the same TPB construct.
