# Supplementary material for: Theory of planned behaviour can help understand processes underlying the use of two emergency medicine diagnostic imaging rules
Source: Implement Sci. 2014 Aug 7;9:88. doi: 10.1186/s13012-014-0088-x (PMC4243777; doi:10.1186/s13012-014-0088-x)
Supplement: Additional file 2: — Description of TPB items from CCHR surveys, arranged by construct. [file 13012_2014_88_MOESM2_ESM.docx]

Appendix 2. Description of TPB items from CCHR surveys, arranged by construct

| **TPB Construct** | **Cronbach’s Alpha** | **Survey Item Descriptions with *Ends of Response Scale*** | **Mean (S.D.)** | **Cronbach’s Alpha if Item Removed** |
| --- | --- | --- | --- | --- |
| Attitude (n = 97) | 0.98 | 1. Managing Patients without CT is: *Bad Practice / Good Practice* | 5.35 (1.50) | 0.98 |
|  |  | 2. Managing Patients without CT is: *Harmful / Beneficial* | 5.03 (1.62) | 0.98 |
|  |  | 3. Managing Patients without CT is: *Negative / Positive* | 5.19 (1.54) | 0.96 |
|  |  | 4. Managing Patients without CT is the: *wrong thing to do / right thing to do* | 5.26 (1.52) | 0.96 |
|  | | | | |
| Subjective Norms  (n = 96) | 0.55 | 1. Most of my Professional Colleagues will Manage Patients without CT: *Definitely No / Definitely Yes* | 4.74 (1.54) | 0.14 |
|  |  | 2. People Important to Me think I should Manage Patients without CT: *Definitely Should Not/Definitely Should ** | 3.54 (1.66) | 0.86 |
|  |  | 3. The Canadian Association of Emergency Physicians would: *Definitely Disapprove/ Definitely Approve* | 5.35 (1.39) | 0.05 |
|  | | | | |
| Perceived Behavioral Control  (n = 98) | 0.71 | 1. Managing Patients without CT is: *Difficult / Easy ** | 4.02 (1.64) | 0.66 |
|  |  | 2. Likelihood that you will be able Manage Patients without CT is: *Very Unlikely / Very Likely* | 5.39 (1.45) | 0.56 |
|  |  | 3. I am confident that I could Manage Patients without CT if I wanted to: *Strongly Disagree / Strongly Agree* | 5.54 (1.31) | 0.60 |
|  |  | 4. Factors outside my control prevent me from Managing Patients without CT: *Strongly Agree / Strongly Disagree ** | 4.24 (1.83) | 0.73 |
|  |  | 5. How much control do you have over Managing Patients without CT: *No Control / Complete Control* | 5.55 (1.18) | 0.67 |
|  | | | | |
| Intention  (n = 97) | 0.98 | 1. I intend to Manage Patients without CT: *Definitely Do Not / Definitely Do* | 5.33 (1.66) | 0.96 |
|  |  | 2. I want to Manage Patients without CT: *Definitely Do Not / Definitely Do* | 5.39 (1.67) | 0.97 |
|  |  | 3. I plan to Manage Patients without CT: *Definitely Do Not / Definitely Do* | 5.36 (1.65) | 0.96 |
|  |  | 4. My Desire to Manage Patients without CT can be described as: *No Desire / Very Strong* | 5.12 (1.42) | 0.98 |

* Polarity of anchors is reversed relative to anchors of other items used for the same TPB construct.
